# Cancerous Disease of the Œsophoagus, with Ulceration into the Bronchi

**Published:** 1867-09

**Authors:** 


### CANCEROUS DISEASE OF THE ŒSOPHAGUS, WITH ULCERATION INTO THE BRONCHI.

Dr. Fisher exhibited an instructive specimen of cancerous disease of the œsophagus, impiclating both lungs.

The patient, aged 52 years, had suffered some time from irritation of the stomach, and difficulty in retaining his food, when, in July, 1866, he sought the advice of Dr. F.

The patient was emaciated, apparently from want of food; for, although his appetite was good, he was unable to swallow. After the passage of a small probang through the obstruction, he was able to swallow liquid, with considerable difficulty, however, for three weeks. Subsequently it was necessary to pass the probang every few days.

During the last five weeks of the patient's life there was increasing difficulty of breathing. There was marked dullness, with crepitant rales; food which he attempted to swallow was coughed up. Patient died early in May, 1867.

An examination revealed the presence of ten ounces of serous fluid in the right thoracic cavity. The œsophagus was attached to both lungs. There was quite a large opening through its walls into the right bronchus, the contiguous portion of the lung being gangrenous. On the left side there was simply a small communication into the bronchus, with scarcely any disease of the substance of the lung.

The lower portion of the œsophagus, throughout its whole circumference, and to an extent of about four inches in length, was indurated, its sides perforated, and its internal diameter contracted to the size of a quill. The microscope showed the structure of the growth to be malignant.

